# Long-Term Effects of a Randomised Controlled Trial Comparing High Protein or High Carbohydrate Weight Loss Diets on Testosterone, SHBG, Erectile and Urinary Function in Overweight and Obese Men

**DOI:** 10.1371/journal.pone.0161297

**Published:** 2016-09-01

**Authors:** Lisa J. Moran, Grant D. Brinkworth, Sean Martin, Thomas P. Wycherley, Bronwyn Stuckey, Janna Lutze, Peter M. Clifton, Gary A. Wittert, Manny Noakes

**Affiliations:** 1 The Robinson Research Institute, Discipline of Obstetrics and Gynaecology, University of Adelaide, Adelaide, South Australia, Australia; 2 CSIRO Food and Nutrition, Adelaide, South Australia, Australia; 3 Freemasons Foundation Centre for Mens Health, University of Adelaide, and South Australian Institute for Health and Medical Research, Adelaide, South Australia, Australia; 4 Division of Health Sciences, University of South Australia, Adelaide, South Australia, Australia; 5 Keogh Institute for Medical Research, University of Western Australia, Perth, Western Australia, Australia; Carolina Urologic Research Center, UNITED STATES

## Abstract

**Introduction:**

Obesity is associated with reduced testosterone and worsened erectile and sexual function in men. Weight loss improves these outcomes. High protein diets potentially offer anthropometric and metabolic benefits, but their effects on reproductive and sexual outcomes is not known.

**Aim:**

To examine the long-term effects of weight loss with a higher protein or carbohydrate diet on testosterone, sex hormone binding globulin, erectile dysfunction, lower urinary tract symptoms and sexual desire in overweight and obese men.

**Methods:**

One-hundred and eighteen overweight or obese men (body mass index 27–40 kg/m^2^, age 20–65 years) were randomly assigned to an energy restricted higher protein low fat (35% protein, 40% carbohydrate, 25% fat; n = 57) or higher carbohydrate low fat diet (17% protein, 58% carbohydrate, 25% fat, n = 61) diet for 52 weeks (12 weeks weight loss, 40 weeks weight maintenance). Primary outcomes were serum total testosterone, sex hormone binding globulin and calculated free testosterone. Secondary outcomes were erectile function as assessed by the International Index of Erectile Function (IIEF) (total score and erectile function domain), lower urinary tract symptoms and sexual desire.

**Results:**

Total testosterone, sex hormone binding globulin and free testosterone increased (P<0.001) and the total IIEF increased (P = 0.017) with no differences between diets (P≥0.244). Increases in testosterone (P = 0.037) and sex hormone binding globulin (P<0.001) and improvements in the total IIEF (P = 0.041) occurred from weeks 0–12 with a further increase in testosterone from week 12–52 (P = 0.002). Increases in free testosterone occurred from week 12–52 (p = 0.002). The IIEF erectile functon domain, lower urinary tract symptoms and sexual desire did not change in either group (P≥0.126).

**Conclusions:**

In overweight and obese men, weight loss with both high protein and carbohydrate diets improve testosterone, sex hormone binding globulin and overall sexual function.

**Trial Registration:**

Anzctr.org.au ACTRN12606000002583

## Introduction

Overweight and obesity are major public health issues that are associated with adverse health problems including cardiovascular disease, diabetes, cancer and poor psychological health [1]. In 2013 overweight and obesity were estimated to be present in over one third (36.9%) of the worlds men [1]. There is an increasing recognition in men of the association between excess weight and erectile dysfunction, reduced sexual desire or enjoyment of sexual activity [2] and lower urinary tract symptoms [3]. Testosterone and its binding protein sex hormone binding globulin (SHBG) are also decreased in obesity [4]. Weight loss increases testosterone and SHBG [5], improves erectile function and sexual desire [2,6–9] and lower urinary tract symptoms (LUTS) [6,7].

There is increasing interest in the optimal diet composition for achieving weight loss and associated metabolic, reproductive or psychological benefits. A recent systematic review reported that a low fat diet, higher in protein and lower in carbohydrate, attenuates loss of lean body mass and is more effective for reducing triglycerides and improving glycemia in comparison to a standard high carbohydrate low fat diet [10]. However, it is unclear whether variations in macronutrient composition associated with these diets, affect testosterone, SHBG, erectile function, sexual function and LUTS independent of any changes in body weight. Short term (10–31 day) studies have reported increases in testosterone or SHBG for higher carbohydrate compared to higher protein weight loss diets [11,12]. However, there is no research assessing these diets during longer-term weight loss or on the clinical outcomes of erectile function, sexual function and LUTS in addition to changes in testosterone and SHBG. We previously reported that long-term higher protein or higher carbohydrate weight loss diets resulted in equivalent improvements in weight, metabolic and psychological outcomes [13,14]. The aim of this current study was, therefore, to examine the long-term effects of weight loss with these diets on testosterone, SHBG, erectile dysfunction, sexual desire and LUTS, in overweight and obese men.

## Methods

### Participants

**Fig 1:**
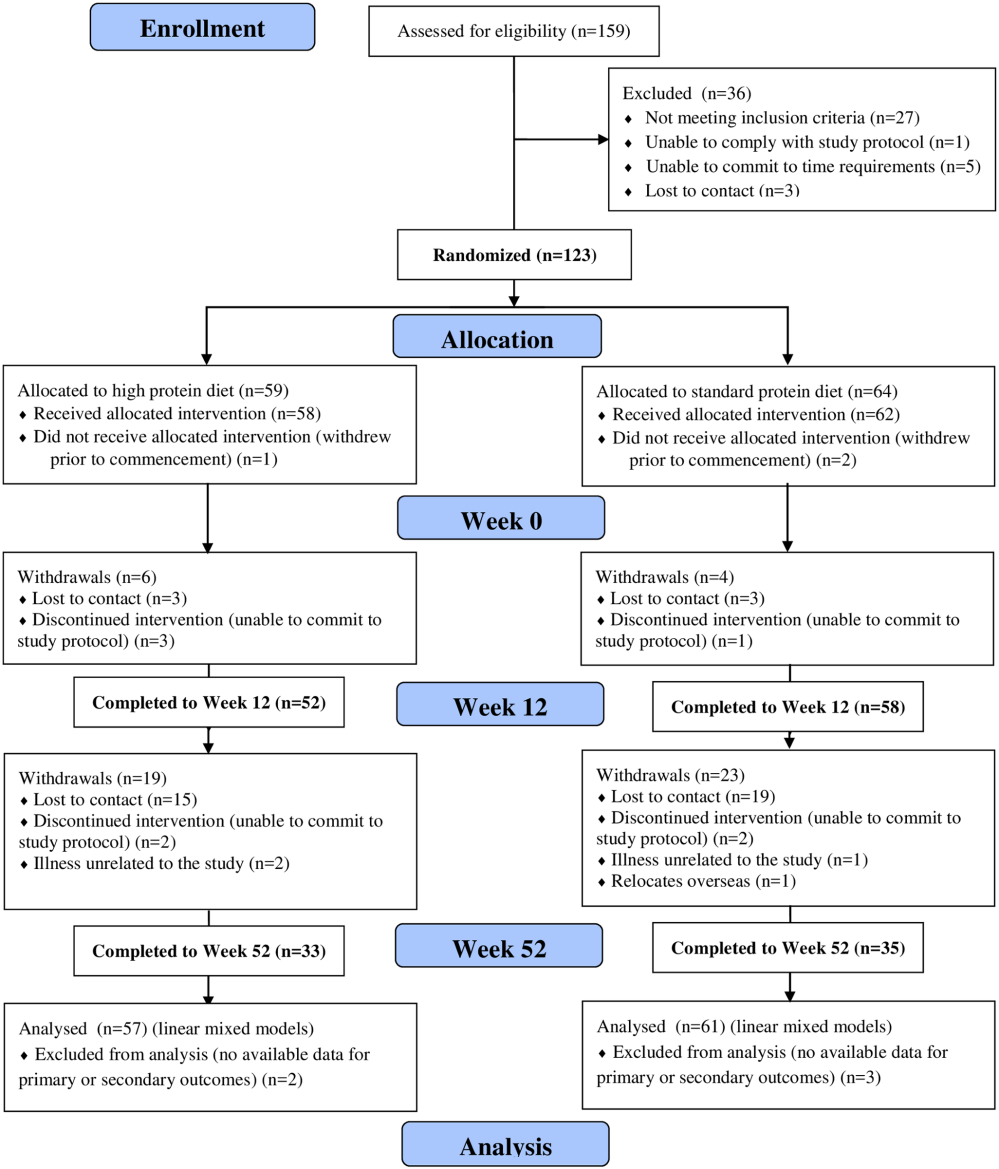
CONSORT diagram.

Overweight or obese males (n = 123) were recruited by public advertisement (Fig 1). Inclusion criteria were body mass index (BMI) 27–40 kg/m^2^ and age 20–65 years and exclusion criteria were diabetes or uncontrolled hypertension; a history of gastrointestinal, renal, coronary, metabolic or hepatic disease or a malignancy and use of hypoglycaemic medication or drugs which affect insulin sensitivity and smoking. This study comprises participants from a larger study [13] (Fig 1) and specifically involved participants who had data available for the primary outcomes of serum total testosterone and SHBG and calculated free testosterone (n = 110) or secondary outcomes of erectile dysfunction, sexual desire and LUTS (n = 118). There were no differences in demographic, anthropometric, metabolic or psychological features between those included or not included in this substudy with the exception of age with those included being older (49.6 vs 40.1 years, p<0.001). The study was approved by the Human Research Ethics committee of the Commonwealth Scientific and Industrial Research Organisation (CSIRO) and all participants provided written informed consent before commencement [13]. The ethics committee approved the study on the 28^th^ February 2005, patient recruitment commenced 2^nd^ March 2005 and follow up completed on 30^th^ June 2006. The study was registered with the Australia New Zealand Clinical Trials Registry (ACTRN12606000002583) on the 3^rd^ of January 2006. The dataset used here was for a study which received ethical approval in 2005 prior to the recommendation of the International Committee of Medical Journal Editors that from July 1, 2005 no trials will be considered for publication unless they are included on a clinical trials registry. Once this recommendation was released, the trial was submitted for registration in a timely manner. The authors confirm that all ongoing and related trials for this intervention are registered with the Australia New Zealand Clinical Trials Registry. S1 Table contains the minimal data set, S2 Table contains the CONSORT checklist and S1 File contains the study protocol.

### Intervention

Participants were block-matched for age and BMI and randomly assigned through computer number generation to the consumption of either a low-fat, isocaloric, energy restricted (7 MJ/day) higher protein (HP) lower glycaemic index diet (35% protein, 40% carbohydrate, 25% fat; n = 57) or higher carbohydrate (HC) lower glycaemic index diet (17% protein, 58% carbohydrate, 25% fat; n = 61) for 52 weeks. The dietary protocol has been previously described in detail [13]. It consisted of a 12 week weight loss (WL) period, where participants met individually with a qualified dietitian at baseline and fortnightly and were providing with key foods representing ~60% of recommended energy intake. This was followed by a 40 week diet maintenance (DM) period with individual dietitian consultations performed monthly. Dietary intake was assessed through the analysis of 3 non-consecutive days (1 weekend day and 2 weeks days) of each 2 week period of diet record data throughout the study, and analysed using a computerised database (Foodworks Professional Edition, version 4, 1998; Xyris Software, Highgate Hill, Australia). For 24 h immediately before the clinical assessments participants conducted a 24-h urine collection for assessment of urinary urea and creatinine as previously described [13]. No specific physical activity was recommended and participants were asked not to modify their lifestyle patterns during the study.

### Primary and secondary outcomes

The primary and secondary outcomes were assessed after an overnight fast at Weeks 0 (baseline), 12 (end of WL phase) and 52 (end of the DM phase). The primary outcomes were serum total testosterone and SHBG and calculated free testosterone which were measured as previously described [6]. The secondary outcomes were erectile function, sexual desire and LUTS. The International Index of Erectile Function (IIEF-15) [15] and the specific erectile function domains were used to assess changes in overall sexual function and erectile function respectively. A score ≥16 on the IIEF erectile function domain was indicative of moderate or severe erectile dysfunction [16]. The Sexual Desire Inventory (SDI) was used to assess the interest of engaging in sexual activity [17]. The International Prostate Symptom Scale (IPSS) was used to separately assess lower urinary tract symptoms (LUTS) relating to storage (increased frequency and/or urgency of micturition, and nocturia) and voiding (incomplete emptying, intermittent and/or weak stream, and straining during micturition) symptoms [18]. Participants were classified as having storage or voiding symptoms as previously described [19] and a score of >3 or 4 were indicative of presence of storage or voiding symptoms. A higher score for IIEF and SDI and a lower score for IPSS indicated improved function. Hypogonadism was defined as a total testosterone of <8 nmol/L or <12 nmol/L if accompanied by a clinical symptom of low testosterone (erectile dysfunction defined as the IIEF erectile function domain <16 indicating the presence of moderate to severe erectile dysfunction symptoms).

### Anthropometric, metabolic and psychological outcomes

Additional outcomes were examined to allow their contribution to changes in primary and secondary outcomes to be assessed. These included height, weight, waist circumference, body composition (total fat mass, total lean mass, body fat % and abdominal fat mass by dual x-ray absorptiometry), seated systolic blood pressure (SBP) and diastolic blood pressure (DBP) (mean arterial pressure (MAP) calculated as [{2/3 x DBP} + {1/3 x SBP}] and lipid profile (total cholesterol, triglycerides, high-density lipoprotein cholesterol [HDL-C], low-density lipoprotein cholesterol [LDL-C], highly sensitive C-reactive protein [hsCRP], insulin, glucose and the homeostasis assessment of insulin resistance [HOMA; fasting insulin x fasting glucose / 22.5] measured as previously described [13]. The profiles of mood states (POMS) component related to depression-dejection (subscale of 15 items) was used as previously described [14] with a higher score indicating a greater degree of mood disturbance.

### Statistical analysis

Two-tailed statistical analyses were performed using Stata (Stata/IC 13.1 for Windows 2014) with statistical significance set at a P-value of <0.05. The normality of the data was assessed using the Kolmogorov-Smirnov test. Data are presented as mean±standard error of the mean (SEM) except where indicated, and log-transformed for analysis if not normally distributed (hsCRP, insulin and HOMA). hsCRP values of >10 mg/L were excluded from the analysis. Cross-sectional data were assessed using t-test or chi-square test. Comparisons between time points were assessed using Wilcoxon test for non-parametric data and linear mixed effects models for parametric data. Linear mixed models were conducted using an unstructured covariance matrix including diet treatment group, time, and their interaction. When the interaction was not significant, it was removed from the model, and the main effect of time was estimated. Separate models were also constructed with inclusion of age, anthropometric, cardiometabolic or psychological variables, androgens or medication status [medication for depression or another psychological condition (n = 13), blood pressure (n = 24) or high cholesterol (n = 40)] or medical history (a prior history of depression, high blood pressure or high cholesterol (n = 71) or adjustment for baseline outcomes. On post-hoc power calculations for the primary outcomes, with n = 57 participants per intervention group, this study had 99% and 80% power (α = 0.05) to detect a difference of 3.41 nmol/L in testosterone and SHBG of 6.21 nmol/L, respectivly.

## Results

### Anthropometric and metabolic outcomes

One-hundred and eighteen participants (HP n = 57, HC n = 61) commenced the intervention, 110 participants completed the 12 weeks WL phase (HP n = 52, HC n = 58) and 68 participants completed the entire 52 week study (HP n = 33, HC n = 35) with no differences in attrition between groups [13]. 32/118 of the participants were overweight (27.1%) and 86/118 of the participants were obese (72.9%) with no significant differences between the treatment groups (p = 0.679). As previously reported, good compliance with both diet interventions was achieved as indicated by no differences between groups for energy intake, lower carbohydrate and higher protein and fat intake and higher twentry-four hour urinary urea for the HP diet compared to the HC diet for the WL and DM phase [13]. As previously reported, there were no significant differences between the treatment groups for baseline anthropometry, metabolic, reproductive or psychological parameters (Table 1). At baseline n = 5 (4.5%) of participants were hypogonadal based on a total testosterone <8 nmol/L, n = 17 (15.6%) of participants were hypogonadal based on a total testosterone <12 nmol/L and the presence of moderate to severe erectile dysfunction symptoms, 28 (24.6%) of participants had moderate to severe erectile dysfunction symptoms, 51 (44.7%) had storage LUTS symptoms and 22 (19.3%) had voiding LUTS symptoms.

**Table 1:** Baseline characteristics of participants.

Outcome measures	HP n = 57	HC n = 61	Mean difference, 95% CI, P-value
Age (years)	50.1±1.2	49.2±1.2	0.84 (-2.5, 4.2), P = 0.622
**Anthropometric**			
Weight (kg)	105.2±1.9	102.6±1.8	2.6 (-2.5, 7.8), P = 0.318
BMI (kg/m^2^)	33.8±0.6	32.7±0.5	1.0 (-0.48, 2.5), P = 0.179
Waist circumference (cm)	111.2±1.4	109.4±1.3	1.8 (-1.9, 5.5), P = 0.345
Total fat (kg)	34.9±1.1	33.2±1.0	1.4 (-1.5, 4.3), p = 0.263
Total lean (kg)	65.5±1.0	64.4±1.1	1.3 (-1.7, 4.2), p = 0.482
Body fat (%)	34.4±0.7	33.8±0.6	0.4 (-1.3, 2.1), p = 0.495
Abdominal fat (kg)	3.1±0.1	2.9±0.1	1.7 (-0.2, 0.5), p = 0.293
**Primary outcomes**			
Testosterone (nmol/L)	13.4±0.6	14.2±0.5	-0.86 (-2.4, 0.66), P = 0.265
Free testosterone (pmol/L)	221.8±10.3	236.1±9.7	-14.1 (-42.3, 13.8), P = 0.316
SHBG (nmol/L)	26.8±1.4	29.1±1.5	-2.4 (-6.4, 1.6), P = 0.244
**Secondary outcomes**			
IIEF total	48.2±2.2	48.3±2.0	-0.09 (-5.9, 5.7), P = 0.974
IIEF-EF	20.9±7.3	21.4±6.8	-0.44 (-3.1, 2.2), P = 0.740
IPSS storage	3.2±0.3	4.1±0.4	-0.96 (-1.9, 0.01), P = 0.052
IPSS voiding	1.8±0.3	2.9±0.4	-1.0 (-2.2, 0.1), P = 0.084
SDI	56.1±2.3	55.3±1.8	0.85 (-4.8, 6.5), P = 0.769
**Covariates**			
Cholesterol (mmol/L)	5.2±0.13	5.3±0.1	-0.09 (-0.43, 0.25), P = 0.594
Triglycerides (mmol/L)	1.7±0.12	1.9±0.1	-0.16 (-0.49, 0.16), P = 0.313
HDL-C (mmol/L)	1.2±0.05	1.3±0.05	-0.05 (-0.18, 0.07), P = 0.409
LDL-C(mmol/L)	3.2±0.1	3.2±0.1	0.04 (-0.27, 0.34), P = 0.817
hsCRP (mg/L)	2.8±0.3	2.7±0.3	0.08 (-0.71, 0.87), P = 0.839
Insulin (mU/L)	12.5±1.2	10.1±0.7	2.4 (-0.36, 5.2), P = 0.087
Glucose (mmol/L)	5.8±0.09	5.9±0.1	-0.04 (-0.36, 0.27), P = 0.782
HOMA	3.3±0.4	2.7±0.2	0.57 (-0.26, 1.4), P = 0.177
SBP (mmHg)	134.5±2.0	135.9±1.6	-1.3 (-6.4, 3.8), P = 0.610
DBP (mmHg)	85.7±1.4	84.2±1.3	1.5 (-2.3, 5.3), P = 0.426
MAP (mmHg)	102.1±1.5	101.4±1.3	0.69 (-3.3, 4.7), P = 0.733
POMS depression	23.6±1.2	23.9±1.0	-0.22 (-3.2, 2.8), P = 0.887

BMI: body mass index, DBP: diastolic blood pressure; EF: erectile function; hsCRP: highly sensitive C-reactive protein; HDL-C: high density lipoprotein cholesterol; HOMA: homeostasis assessment of insulin resistance; HC: higher carbohydrate; HP: higher protein; IIEF: international index of erectile dysfunction; IPSS: international prostate symptom score; LDL-C: low density lipoprotein cholesterol; MAP: mean arterial pressure; POMS: profile of mood states; SDI: sexual desire inventory; SBP: systolic blood pressure

Data are presented as mean±SEM and were analysed by independent t-test with the between subject factor of diet

There were no significant differences between the treatment groups for the change in weight over the intervention duration (P = 0.924) with both the HP and HC achieving a significant weight loss from week 0–12 (9.1±0.6, p<0.001 vs 8.8±0.6 kg, p<0.001) and week 0–52 (10.8±1.2, p<0.001 and 10.3±1.2, p<0.001). Similarly, there was no differential effect of diet for the change in anthropometric, metabolic or psychological outcomes over the study duration [13] (Table 2). On assessment of all participants combined, significant decreases occurred for all variables from Weeks 0–12 with the exception of POMS depression and from Weeks 0–52 with the exception of glucose, HOMA and POMS depression (Table 2).

**Table 2:** Change in outcomes with weight loss intervention for all participants combined.

Outcome measures	0–12 weeks	12–52 weeks	0–52 weeks
Weight (kg)	-8.9±0.4, P<0.001	-1.6±0.6, P = 0.034	-10.5±0.8, P<0.001
BMI (kg/m^2^)	-2.8±0.1, P<0.001	-0.6±0.2, P = 0.018	-3.4±0.3, P<0.001
Waist circumference (cm)	-10.4±0.4, P<0.001	-0.7±0.5, P = 0.700	-11.0±0.7, P<0.001
Total fat (kg)	-6.1±0.3, P<0.001	-1.9±0.6, P = 0.008	-8.0±0.6, P<0.001
Total lean (kg)	-2.6±0.3, P<0.001	-0.09±0.3, P<1.000	-2.7±0.4, P<0.001
Body fat (%)	-3.7±0.3, P<0.001	-1.3±0.5, P = 0.014	-5.0±0.5, P<0.001
Abdominal fat (kg)	-0.7±0.03, P<0.001	-0.1±0.05, P = 0.116	-0.8±0.06, P<0.001
Cholesterol (mmol/L)	-0.7±0.1, P<0.001	+0.4±0.1, P<0.001	-0.4±0.1, P<0.001
Triglycerides (mmol/L)	-0.5±0.1, P<0.001	+0.01±0.1, P<1.000	-0.4±0.1, P<0.001
HDL-C (mmol/L)	-0.02±0.02, P<1.000	+0.1±0.02, P<0.001	+0.1±0.02, P<0.001
LDL-C (mmol/L)	-0.5±0.1, P<0.001	+0.2±0.1, P = 0.003	-0.3±0.1, P<0.001
hsCRP (mg/L)	-0.5±0.1, P = 0.003	-0.4±0.2, P = 0.087	-0.9±0.2, P<0.001
Insulin (mU/L)	-4.4±0.6, P<0.001	+0.3±1.1, P = 1.000	-4.1±1.3, P = 0.005
Glucose (mmol/L)	-0.3±0.1, P<0.001	+0.1±0.1, P = 0.600	-0.2±0.1, P = 0.115
HOMA	-1.3±0.2, P<0.001	+0.4±0.5, P<1.000	-0.9±0.5, P = 0.243
SBP (mmHg)	-3.9±1.4, P = 0.015	-2.0±1.6, P = 0.637	-5.9±1.4, P<0.001
DBP (mmHg)	-4.2±1.2, P = 0.002	-2.8±1.3, P = 0.090	-7.0±1.1, P<0.001
MAP (mmHg)	-4.2±1.2, P = 0.003	-2.6±1.3, P = 0.158	-6.7±1.2, P<0.001
POMS depression	-0.2±0.4, P<1.000	+0.04±0.5, P<1.000	-0.2±0.7, P<1.000

BMI: body mass index, DBP: diastolic blood pressure; hsCRP: high sensitivity C-reactive protein; HDL-C: high density lipoprotein cholesterol; HOMA: homeostasis model assessment of insulin resistance; LDL-C: low density lipoprotein cholesterol; MAP: mean arterial pressure; POMS: profile of mood states; SBP: systolic blood pressure

Data are presented as mean±SEM and were analysed by linear mixed models with the fixed factors time, diet and time-by-diet

### Primary outcomes—Total testosterone, calculated free testosterone and SHBG

**Fig 2:**
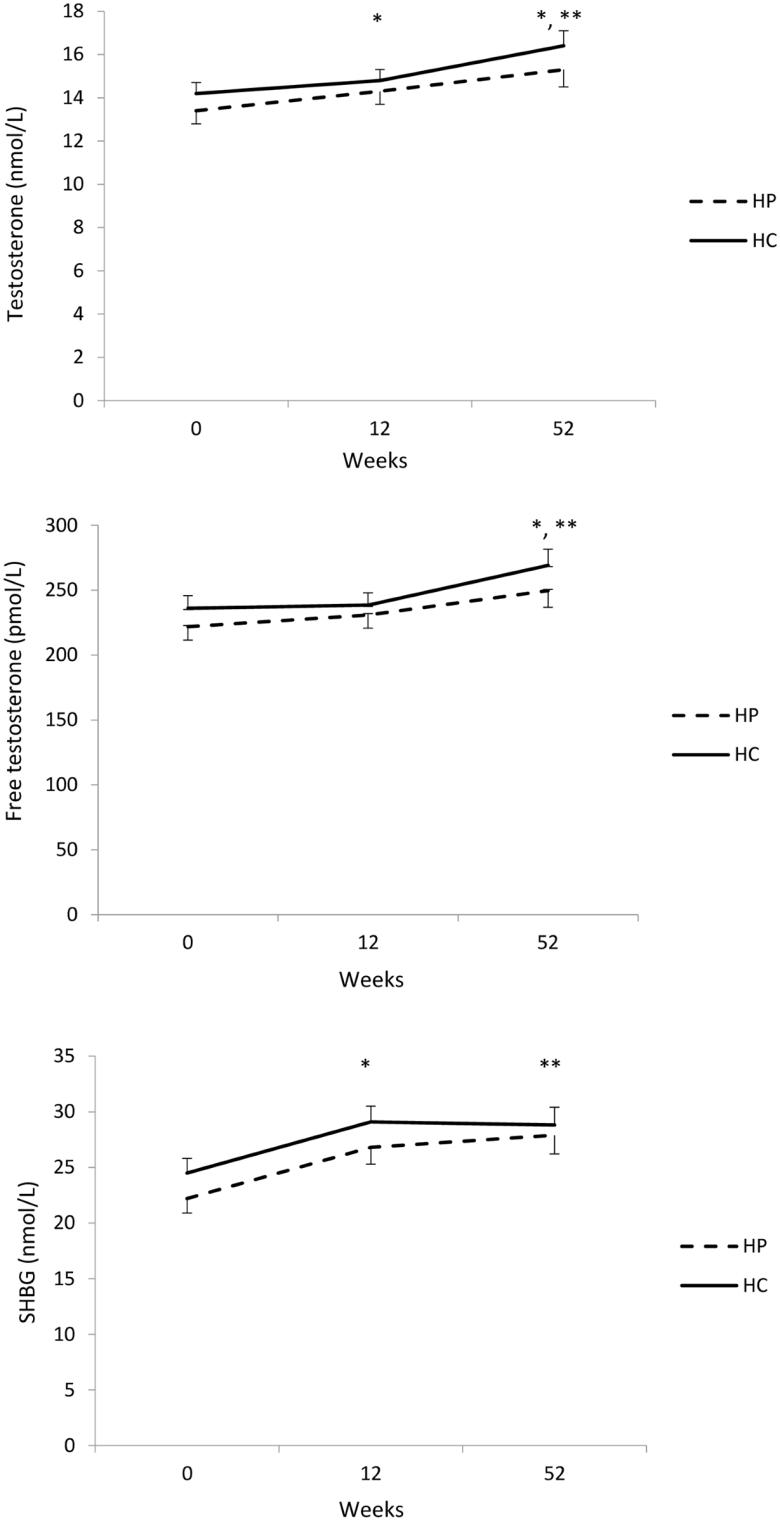
Changes in testosterone, free testosterone and sex hormone binding globulin (SHBG) with a higher protein (HP, n = 57) or higher carbohydrate (HC, n = 61) diet. Data are presented as mean±SEM and were analysed by linear mixed models with the fixed factors time, diet and time-by-diet. * significant change from preceding time point for both interventions (p<0.05). ** significant change from week 0–52 for both interventions (p<0.05).

There was a significant change in serum total testosterone and SHBG and calculated free testosterone over the intervention duration (P<0.001) (Fig 2). Testosterone increased both from Weeks 0–12 (0.68±0.30 nmol/L, P = 0.037) and Weeks 12–52 (1.5±0.4 nmol/L, P = 0.002) with an overall increase from Weeks 0–52 (2.0±0.4 nmol/L, P<0.001). Free testosterone increased significantly from week 12–52 (25.0±6.9 pmol/L, p = 0.002) with an overall increase from week 0–52 (30.5± 7.1 pmol/L, p<0.001). SHBG increased from Weeks 0–12 (4.6±0.5 nmol/L, P<0.001) but did not change from Weeks 12–52 (0.41±0.62 nmol/L, P<1.000) with an overall increase from Weeks 0–52 (5.0±0.7 nmol/L, P<0.001). There was no differential effect of diet on the changes in testosterone (P = 0.670), free testosterone (P = 0.630) and SHBG (P = 0.508).

### Secondary outcomes—Erectile function, sexual desire and lower urinary tract symptoms

There was a significant change in IIEF over the intervention duration (P = 0.017) with an increase from Weeks 0–12 (P = 0.041) but no differential effect of diet on these variables (Table 3). There was no significant change in the erectile function domain of the IIEF, SDI or storage or voiding LUTS as measured by the IPSS over the intervention in either diet group (Table 3) for all participants combined.

**Table 3:** Change in erectile function, lower urinary tract symptoms or sexual desire with a high protein or high carbohydrate diet.

Outcome measures		Week			P-value		
		0	12	52	Time	Diet	Time-by-diet
IIEF Total	HP	48.2±2.1	51.4±1.9*	49.4±2.2	0.017	0.760	0.285
	HC	48.5±2.0	50.0±1.8*	52.2±2.1			
IIEF-EF	HP	20.9±0.9	22.6±0.9	21.8±1.0	0.126	0.780	0.329
	HC	21.5±0.9	22.0±0.8	22.8±1.0			
IPSS Storage	HP	3.2±0.4	2.9±0.4	3.1±0.5	0.649	0.041	0.871
	HC	4.0±0.3	4.0±0.3	4.1±0.4			
IPSS Voiding	HP	1.8±0.4	2.1±0.5	1.8±0.6	0.363	0.055	0.356
	HC	2.8±0.4	3.0±0.5	3.6±0.6			
SDI Total	HP	56.2±2.0	55.6±2.1	55.8±2.2	0.806	0.701	0.794
	HC	54.5±2.0	54.5±2.0	55.6±2.1			

EF: erectile function; HP: Higher protein, low fat diet (n = 57); HC: Higher carbohydrate low fat diet (n = 61); IIEF: international index of erectile dysfunction; IPSS: international prostate symptom score; SDI: sexual desire inventory

Data are presented as mean±SEM and were analysed by linear mixed models with the fixed factors time, diet and time-by-diet

* significant change from preceding time point (P<0.05)

On consideration of the men with presence or absence or moderate to severe erectile dysfunction symptoms at baseline, there was a significant time-by-IIEF erectile function effect (P<0.001). The men with no symptoms or mild erectile dysfunction symptoms had no changes in the IIEF erectile function domain with time while the men with moderate to severe erectile dysfunction symptoms had a significant increase in the IIEF erectile function domain from trial entry to 12 weeks (6.0±1.0, P<0.001) and trial entry to 52 weeks (4.6±1.3, P = 0.002). On consideration of the men with the presence of voiding or storage LUTS symptoms, there was a significant time-by-storage LUTS (P<0.001) and time-by-voiding LUTS effect (P<0.001). There was a decrease in storage LUTS from trial entry to week 12 for those with storage LUTS symptoms at baseline (-1.2±0.3, P<0.001) but an increase for those without storage LUTS symptoms at baseline (0.67±0.2, P = 0.022). There were no differences in the change over time for voiding LUTS for either men with or without voiding LUTS symptoms.

All time by treatment results were maintained on adjustment for age, medical conditions or medication status or changes in anthropometric, metabolic (lipid profile, hsCRP, insulin, glucose, HOMA or blood pressure) or psychological (POMS depression) outcomes or for changes in testosterone, free testosterone or SHBG with the intervention outcomes with the intervention with the exception of the change in SHBG which was removed on adjustment for waist circumference and the change in IIEF which was removed on adjustment for weight, waist circumference, total fat mass, total lean mass, % body fat or total abdominal fat.

## Discussion

These data confirm prior reports in overweight and obese men that weight loss increases testosterone and SHBG [5] and improves overall sexual function [2]. Weight loss did not alter erectile function, sexual desire or lower urinary tract symptoms for all participants combined. An effect of weight loss was observed for participants based on their baseline erectile dysfunction and lower urinary tract symptoms such that only those with moderate to severe erectile dysfunction or the presence of lower urinary tract symptoms related to storage had improvements with weight loss. We report here for the first time that a long-term isocaloric high protein or carbohydrate weight loss diet had similar effects on these outcomes.

Previous research has reported that increasing the carbohydrate-to-protein ratio in reduced energy diets either increases [11,12] or does not alter [20] total testosterone [11,12] and SHBG [12]. Cross-sectional studies have also reported that an increased protein intake is associated with decreased lower urinary tract symptoms [21] potentially related to altered testosterone and SHBG levels [12] or supressed sympathetic nervous system activity [22]. While this suggests a high protein weight loss diet may improve our primary and secondary outcomes to a greater extent than a high carbohydrate weight loss diet, we did not observe this in our current study. We note that the limited prior human research is generally in acute (10–31 day) feeding studies [11,12,20]. Our findings are also in agreement with no differences in testosterone, SHBG, erectile function, lower urinary tract symptoms or sexual desire following a higher protein or meal replacement weight loss diet [7], although the degree of energy restriction and weight loss was different between these two approaches. This suggests that weight loss with either a higher protein or a higher carbohydrate are feasible options to improve reproductive function in overweight or obese men.

Our finding that weight loss increased total testosterone and SHBG is consistent with a recent meta-analysis of 13 studies with a mean weight loss of 9.8% and a maximum follow-up time of 104 weeks [5]. Our improvements in the total IIEF with weight loss and for the IIEF erectile function domain for those with moderate to severe erectile function symptoms at baseline are consistent with prior research reporting improvements in erectile function with weight losses of 4.7–15 kg over 8 weeks to 2 years [6–9,23,24]. Our observed improvements are also consistent with recommended minimally clinically important differences of 4 units for the erectile function domain [25], highlighting the clinical relevance of our intervention. Of interest, these improvements have been reported in participants both with [8] or without erectile dysfunction [23] while here we observed improvements only for those with baseline erectile dysfunction. Decreased testosterone may be associated with reduced nitric oxide production, erectile dysfunction and worsened sexual function [26–28] and increased testosterone following weight loss may thus be associated with improved erectile function although the threshold for these effects is not clear. However, we observed no mediating effect of testosterone on changes in the IIEF or the IIEF erectile dysfunction domain in this study. We also extend the prior literature to report that the benefits of weight loss on testosterone SHBG and overall sexual function for all participants and erectile function for those with baseline erectile dysfuncton was independent of the potential moderating effect of cardiometabolic and psychological status including consideration of the effect of surrogate markers of insulin resistance, dyslipidaemia, inflammation, glycaemia and depressed mood while the change in erectile function were ameliorated on adjustment for changes in anthropometry. This suggests that the positive effect of our intervention on erectile function is primarily related to changes in weight.

The observed increases in testosterone and SHBG occurred with short-term acute weight loss (9 kg over 12 weeks) and were sustained during weight maintenance up to 52 weeks. However, the increase in testosterone was modest for the acute weight loss phase (0.68 nmol) with a more marked increase experienced during longer-term weight maintenance (1.5 nmol) that occurred in the absence of any increase in SHBG. It is unclear whether these modest changes in acute weight loss are sufficient to result in substantive effects at the tissue level. The reason for this delayed increase is testosterone is unclear. Given that sexual activity increases testosterone [29], it may be occurring as a consequence of the improvement in overall sexual function with acute weight loss. It may also indicate the removal of the dampening effect on the HPG axis of excessive estrogen via peripheral aromatisation, although neither estradiol or luteinising hormone (LH) was measured in this study. Alternatively, the energy deficit may supress an increase in testosterone consistent with prior findings increases in testosterone following restoration of nutrient intake [30] This may indicate longer-term interventions are required for larger increases in testosterone.

However, the improvement in overall sexual function occurred only with short-term weight loss. This is in contrast to previous reports of further improvements in erectile function during long-term follow-up (up to 52 weeks of weight maintenance following 8 weeks of weight loss) [7], improvements following a 8.9–15 kg weight loss over 1–2 years [8,9,24] and sustained improvements following bariatric surgery up to 2 years [31]. It is not clear why overall sexual function improvements were not maintained here since the degree of weight loss (10.5 kg) is comparable to the other dietary interventions (10–15 kg). However, in these prior studies weight loss was often achieved as part of a multidisciplinary lifestyle intervention combining diet and physical activity with improvements in erectile function significantly associated with physical activity or fitness [8,9]. Given the association of physical activity with improved endothelial function through improving mechanisms such as nitric oxide production [32], a multidisciplinary approach including physical activity may therefore be required for optimising erectile function. This is also an important consideration for sustaining improvements in clinical outcomes long-term. It is also possible that the lack of a sustained improvement in overall sexual function is related to the lack of further improvements in waist circumference and surrogate markers of insulin resistance from week 12–52 which are turn are likely related to weight being maintained rather than further reduced during this time.

In agreement with prior research, we report here no changes in sexual desire [33] or lower urinary tract symptoms [34] following weight loss for all participants combined. Our findings are in contrast to prior reports [6,7,35,36] where improvements in the SDI or IPSS occurred following weight losses of 2-6-12.3 kg over 8–52 weeks. We note that an improvement in the storage LUTS score occurred for those with the presence of features of lower urinary tract symptoms relating to storage symptoms at baseline. This likely indicates that the beneficial effect of weight loss is observed only when abnormal function is present, Improvements in erectile function would also be expected to occur in association with improvement in sexual desire and lower urinary tract symptoms as previously reported [6]. This could be related to improvements in the underlying mechanisms common to all of these conditions including altered endothelial function, pelvic blood flow or autonomic system function [37]. The reason for our discrepant findings are unclear. One prior study assessed populations with a large proportion of type 2 diabetes and abdominal obesity and reported greater improvements in IPSS for the non-diabetic subjects [6]. Here our baseline SDI and IPSS were more similar to the participants with diabetes (44.1 and 5.8) than those without diabetes (71.2 and 18.8). While our study did not specifically recruit individuals with type 2 diabetes, our population here may be more metabolically compromised who may have required a greater degree of weight loss to achieve improvements in sexual function or lower urinary tract symptoms.

We report here the first randomised controlled trial assessing the effect of modifying the macronutrient content of a weight loss diet on testosterone, SHBG, overall sexual function, erectile function, lower urinary tract symptoms and sexual desire. We also report these effects over a long-term intervention (1 year), thus increasing the clinical applicability of these findings. The strengths of this study include the consideration of potential confounding factors including medication status, medical history or concurrent metabolic, androgen or psychological outcomes. We note weaknesses here including the use of a surrogate marker of depression as the POMS depression subscale as opposed to validated questionnaires or clinician-verified diagnoses. Consideration of additional potential mediators such as physical activity, endothelial function, autonomic function and luteinising hormone would also be of interest in future research and further studies should examine these outcomes concurrently to better understand the underlying mechanisms for the observed effects. We also reported a secondary analysis of a study which was initially powered on weight changes between the two diet groups, however post-hoc analyses revealed we were sufficiently powered for the primary outcomes reported in this analysis. We also report here a relatively high dropout rate. While this may limit the generalisability of these findings, this is comparable to other long-term dietary interventions [38,39]. We note that further research is also warranted assessing the effects of the diet compositions studied here on a a range of health dimensions or domains in addition to sexual function including vascular and renal function [40,41].

In conclusion, after 1 year, energy restricted low fat diets either higher in protein or carbohydrate similarly improved testosterone, SHBG and overall sexual function in overweight and obese men but did not improve erectile function, sexual desire or lower urinary tract symptoms. These findings suggest that overweight and obese men can obtain improvements in sexual function from caloric restriction induced weight loss, irrespective of the dietary protein-to-carbohydrate ratio.

## Supporting Information

S1 File Study protocol. (DOC)

S1 Table Minimal data set. (XLS)

S2 Table CONSORT checklist. (DOC)
