# Supplementary material for: Long-Term Effects of a Randomised Controlled Trial Comparing High Protein or High Carbohydrate Weight Loss Diets on Testosterone, SHBG, Erectile and Urinary Function in Overweight and Obese Men
Source: PLoS One. 2016 Sep 1;11(9):e0161297. doi: 10.1371/journal.pone.0161297 (PMC5008754; doi:10.1371/journal.pone.0161297)
Supplement: S1 File — (DOC) [file pone.0161297.s001.doc]

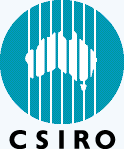


**RED MEAT AND HUMAN NUTRITION:**

**Diet, weight loss, bowel health and total wellbeing in men**

**Human Ethics Committee Approval Number: 04/29**

**Project Protocol**

**Objectives**

**Evaluate whether weight loss on a higher protein-high red meat dietary pattern similar to the CSIRO Total Wellbeing Diet (TWD) compared to a high carbohydrate pattern improves**

- - - ***Cardiovascular Risk***
    - ***Folate and Vitamin B12 Status***
    - ***Physical Performance and Exercise Tolerance***
    - ***Sexual Function***
    - ***Psychological Wellbeing***
    - ***Safety Markers***

# RESEARCH PLAN

## PARTICIPANTS AND STUDY DESIGN

A subset of 60 of the 120 overweight males with insulin resistance will be recruited for this study by public advertisement. Exclusion criteria include: use of any form of drug therapy or medication or dietary supplements on a regular basis that may interfere with bowel function (e.g. laxatives, antibiotics, anti-diarrhoeals or probiotics), consumption of any over-the-counter medication that could interfere with the study, participation in a bowel health study or in any study of an experimental drug within 30 days of commencement of the study or a history or presence of gastrointestinal, renal or hepatic disease of any cause. Full inclusion/exclusion criteria on pg 22.

Participants will be randomly assigned to either a high protein or high carbohydrate diet (table 1). They will undertake these dietary patterns during a 12 week weight loss (WL) period followed by a 40 week diet maintenance (DM) period (figure 1). Faecal water (aqueous phase isolated from faecal samples) and rectal biopsy samples will be taken at weeks 0, 12 and 52 (figure 1). Participants will meet with a dietician monthly to help ensure maximum dietary compliance.

|  | **HIGH PROTEIN** | **HIGH CARBOHYDRATE** |
| --- | --- | --- |
| **PROTEIN (%E)** | 35 | 17 |
| **CARBOHYDRATE (%E)** | 40 | 58 |
| **FAT (%E)** | 25 | 25 |

**TABLE 1.** Macronutrient breakdown of diets

**%E** = percentage of total daily energy

**Secondary Objectives**

- To evaluate whether weight loss on a higher protein-high red meat dietary pattern similar to the CSIRO Total Wellbeing Diet (TWD) compared to a high carbohydrate pattern improves
  - - *Cardiovascular Risk Factors*
      - *weight loss*
      - *changes in body fat distribution (DEXA)*
      - *fasting blood pressure*
      - *fasting lipids, insulin and glucose*
      - *fasting folate, homocysteine, C reactive protein*
    - *Folate and Vitamin B12 Status*
    - *Physical Performance and Exercise Tolerance*
    - *Sexual Function*
    - *Psychological Wellbeing*
    - *Safety Markers*

**Study Design**

Parallel study consisting of a 12 week intensive weight loss phase and a 40 week follow-up dietary maintenance period with interim monthly visits.

Figure 1. shows a schematic representation of the study design

**Figure 1.**

**Schematic representation of study design (n=120 participants)**

Participants:

120 male overweight/obese participants will be selected for the study with the following criteria:

Inclusion Criteria

1. Male
2. Be aged 20-65 years
3. At least one CVD risk factor other than obesity
4. Participants must understand the procedures involved and agree to participate in the study by giving full informed, written consent.
5. No abnormality of clinical significance on medical history.(i.e. life-threatening cancer, liver or metabolic disease or cardiovascular problems - unstable angina, previous incidence of myocardial infarction, stroke or heart irregularity).
6. BMI 27-40kg/m2
7. No history of metabolic or coronary disease.
8. Not have type 1 or type 2 Diabetes (self reported)
9. Be available for the duration of the study

Exclusion criteria

1. Use of any form of drug therapy or medication or supplements on a regular basis that may interfere with bowel function (e.g. laxatives, antibiotics, anti-diarrhoeals or probiotics)
2. Consumption of any over-the-counter medication that in the opinion of the investigator could interfere with the study.
3. History or presence of gastrointestinal, renal or hepatic disease of any cause.
4. The following items if taken need to be kept stable during the study: corticosteroids, diuretics, B-blockers, fish oil supplements, cholesterol lowering agents
5. Hypoglycaemic medication & drugs which affect insulin sensitivity
6. History of heavy alcohol consumption (> 5 STD drinks/day)
7. Volunteer unable to cease alcohol consumption for study duration
8. Widely fluctuating exercise patterns
9. Frequent dining out (> 2X/week and unable to cease)
10. Inability to prepare meals or meet diet requirements
11. Extended absences due to travel or other commitments
12. Person considered by the investigator to be unwilling, unlikely or unable to comprehend or comply with the study protocol and restrictions.

Participants will be blocked for BMI and age and randomly assigned to one of 2 diet interventions:

- - Higher Protein High Red Meat Diet (35%P,25%F, 40%C) 7000KJ
  - High Carbohydrate Diet Low Red Meat (20%P, 25%F, 55%C) 7000KJ

Some adjustments in kilojoules for individuals will be necessary to achieve a deficit of 2000-4000KJ per day corresponding to 0.5-1.0kg weight loss per week.

**Dietary Intervention:**

Participants will attend individual consultations with a dietitian who will prescribe the appropriate kilojoule level for the individual and provide instruction on the structured eating plan and dietary requirements, method for recording food intake and need for compliance. Participants will be issued with digital kitchen scales to weigh food. Two cups or more of low carbohydrate vegetables per day will be encouraged. A range of additional low-energy foods will be allowed. Four standard serves of alcohol will be permitted per week. Each 2 weeks until 12 weeks, participants will attend the Clinical Research Unit and will be supplied with key foods consistent with their allocated diet to encourage compliance. Each supply will provide approximately 60% of projected total energy intake days and will be isocaloric between groups.

The fibre content and fatty acid profile of diets will be planned to be the same between diets. Checklists of all foods consumed will be completed daily and three day weighed food records will be analyzed in each 2-week period. Advice on physical activity will be consistent with a recommendation to increase physical activity to at least 30 minutes three times per week and to document these occasions in their daily checklist.

At the conclusion of the 12 week intensive period, participants will be asked to continue the general principles of the dietary pattern they were assigned to and will return on a monthly basis for dietary counseling until the completion of the 1 year study. Assessment of food intake at baseline and at 6 and 12 months will be performed using a validated food frequency questionnaire by The Anti Cancer Council of Victoria which has been validated by Hodge et al 2000 as well as by ourselves in shorter term intervention trials (Xie Xinying et al 2004).

*Prescriptive food composition of test diets*

|  | **High Protein High Red Meat** | **High Carbohydrate Low Red Meat** |
| --- | --- | --- |
| Cereal | 50g high fibre cereal | 50g cereal |
| Milk | 500ml/Day (1.5% fat) | 250ml/Day (1.5% fat) |
| Low Fat Yogurt | 200g | Nil |
| Lean Meat/Poultry/Fish | 250 grams lean beef/ lamb > 6 times/week  + extra 50g other protein food daily (lunch) | 100gram chicken/pork/fish  (>6 times/week)  red meat < 1/week  + extra 30g other protein food daily (lunch) |
| Fresh Fruit | 300g | 450g |
| Pasta/Rice (or potato equivalent) | NIL | 70g dry weight |
| Salad | ½ cup | ½ cup |
| Vegetables | (at least 2.5 Cups) | (at least 2.5 Cups) |
| Oil/spread | 20g | 25g |
| Bread Wholegrain | 105g | 140g |
| Cheese | NIL | 30g |
| Wine or equivalent  (optional) | 500ml/week | 500ml/week |

**SECONDARY OUTCOMES PROTOCOL**

***Cardiovascular risk and body composition***

- ***Body height and mass and composition****.* Body height will be measured using a stadiometer (SECA, Hamburg, Germany) and body mass will be measured using calibrated electronic digital scales (Mercury, AMZ 14, Tokyo, Japan). Body composition will be determined by whole-body dual X-ray absorptiometry (DEXA; Lunar Prodigy, Lunar Radiation Corporation, Wisconsin, USA) with a coefficient of variation of 2.3 ± 0.9% for total fat mass and 2.1 ± 0.4% for total lean mass.
- ***Blood measurements****.* Fasting plasma and serum will be collected, and stored at -80^0^C until the end of the study. Serum lipids and glucose will be measured in one run at the end of the study on a Roche Hitachi analyser using standard Roche enzymatic kits (Roche Diagnostics Co. IN, USA). HDL-C will be measured after PEG 6000precipitation of apoB containing lipoproteins. Serum insulin will be measured using a radioimmunoassay kit (Pharmacia & Upjohn Diagnostics AB, Uppsala, Sweden). C-reactive protein will be measured using an enzymatic kit (Roche, Indianapolis, IN, USA), on a Hitachi auto analyzer (Roche, Indianapolis, IN, USA).

***Vitamin B12 Folate And Homocysteine Status***

Measures for homocysteine, folate, B12 will be outsourced and measured in a certified commercial laboratory - Institute of Medical and Veterinary Science, (Adelaide, South Australia). Holotranscobalamin will be measured using a competitive radiobinding assay (Axis-Shield.

***Physical Performance/Exercise Tolerance***

- ***Measurement of maximal oxygen consumption and response to submaximal exercise****.*

Maximal oxygen consumption (V_max_) will be measured during a graded incremental treadmill exercise test to symptom-limited exhaustion, according to modified Bruce protocol (American College of Sports Medicine, 2000). Participants will be encouraged to continue to exercise until they reach volitional exhaustion or the presence of cardiorespiratory symptoms or abnormalities that contraindicate exercise continuation. The highest oxygen uptake achieved over 30 seconds will be taken as V_max_. After cessation of exercise, participants will be placed supine as soon as possible and recovery data will be obtained.

A 12-lead electrocardiogram (ECG) and heart rate will be measured continuously before exercise, during exercise and for 2 minutes of recovery to detect rhythm disturbances and any sign of ischemia. Blood pressure will be measured manually during the last 30 s of each exercise stage using a sphygmomanometer. Participants will report their rating of perceived exertion (RPE) on the 15-point Borg scale at the end of each workload and at the cessation of exercise. The reason to end the test will also be recorded. Measurements of oxygen uptake (V) and carbon dioxide (VC) will be continuously recorded as 30 second averages before exercise, throughout the incremental exercise test and during the 2-min post-recovery period using an exercise calorimetry system (COSMED K4, Cosmed, Italy), which will be pre-calibrated with commercially-produced known gas mixtures. Participants will breathe through a tightly fitted face-mask and a low-resistance, non-rebreathing respiratory value (Hans Rudolph 2700 series, Kansas City, USA), attached to the metabolic system containing a flow module that will be calibrated with a 3 L syringe prior to each test.

Exercise testing will be terminated based on criteria delineated by the American College of Sports Medicine (American College of Sports Medicine, 2000): if the participant is unable to continue because of symptoms such as fatigue, dyspnea, or chest or leg pain (a symptom-limited test); physical signs including a decrease in systolic blood pressure of >10 mmHg between consecutive stages; exercise systolic pressure ≥ 250 mmHg; an attainment of maximal heart rate, or a decrease of heart rate; or development of significant ECG abnormalities (≥1.0mm depression of resting ST-segment, arrhythmias, ventricular ectopy, or conduction defects); technical difficulties, or non-corporation by the participant.

Exercise tolerance and the metabolic response to exercise resulting from dietary manipulation will be assessed with V_peak_ and lactic acidosis threshold (LAT) determined according to the V-slope method of breakpoints in the relationship between V and VC (77). Participants who fail to achieve ≥85% of their age-predicted maximal heart rate on the maximal exercise test will be excluded from analysis.

- ***Assessment of local muscular strength, power and endurance.***

Peripheral muscular strength, power and endurance (fatigue) will be assessed using an Isokinetic dynamometer (Kin-Com 125AP, Chattecx Corporation, Tennessee, USA) for both the dominant and nondominant lower extremities. Isometric muscle strength will be determined by the peak torque achieved from 3 maximal isometric knee extension contractions with the tested leg positioned at 90^o^; each effort will be separated by 2 minutes of recovery. Muscle power and endurance will be assessed from 30 consecutive maximal-effort concentric knee extension/flexion manoeuvres performed at a pre-set angular velocity of 3.14 rads/s^-1^ (180 degress/sec^-1^). Local muscular endurance in the quadriceps and hamstrings will be assessed by the percent reduction (fatigue index) in peak torque (and work) between the first 5 repetitions and the last 5 knee extension and flexion contractions in a series of 30 maximal voluntary concentric contractions with an angular velocity of 180 degree·sec^-1^. The slope will also be determined via linear regression by plotting the work values for each repetition across the 30 contractions for each subject to quantify the rate of decrease in quadriceps and hamstring work during the exercise bout. Prior to these testing procedures, participants will complete a warm-up by performing 9–10 submaximal familiarization repetitions. All participants will be familiarised with the testing protocol prior to the initial baseline test. Kin-Com measurements are repeatable (e.g., repeated loading and unloading of a strain gauge) and accurate to known weights (e.g., intraclass correlation coefficient [ICC] = .99) in static testing. Reliability of concentric and eccentric torque measurements on the Kin/Com can range from 0.93 to 0.98 for both slow (30 deg/sec) and fast (180 deg/sec) speeds among healthy participants.

***Psychological Wellbeing***

- ***Psychological tests.*** The Profile of Mood States (POMS), the General Well-Being Schedule (GWBS), the Beck Depression Inventory (Centre for Cognitive Therapy, Philadelphia), the Spielberger State-Trait Anxiety Inventory (Mind Garden, Palo Alto, CA) and the Fatigue severity scale questionnaires will be used to assess changes in mood at baseline and every 2-weekly interval. These questionnaires are all established instruments used for the psychological assessment of different mood state characteristics (Fazio 1978; McNair, et al. 1971; McDowell et al. 1996; Beck et al. 1988; Krupp et al. 1989; Shumaker et al. 1990; Spielberger et al. 1983) and have been used repeatedly in the scientific literature to examine changes in psychological state to an experimental intervention, including dietary manipulations.

***Safety Markers***

Urinary urea and creatinine will be measured in one run on a Hitachi auto analyzer (Roche, Indianapolis, IN, USA) at the end of the study.

Urinary deoxypyridinoline and pyridinoline will be measured using high performance liquid chromatography and expressed per mmol creatinine.

All the outcome measures are summarized below.

**OUTCOME MEASURES:**

***BOWEL HEALTH (PRIMARY)***

- - - *faecal samples for markers of bowel health and colon cancer risk (0, 12, 52 weeks)*
    - *faecal water cytotoxity/genotoxicity (0, 12, 52 weeks)*
    - *Faecal weight, short chain fatty acids (0, 12, 52 weeks)*
    - *rectal biopsy to assess genome damage in colonocytes (0, 12, 52 weeks)*
    - *blood sample for lymphocytes (0, 12, 52 weeks)*

***CARDIOVASCULAR HEALTH***

- - - *weight loss*
    - *changes in body fat distribution (DEXA) (0, 12, 52 weeks)*
    - *fasting blood pressure (0, 4, 8, 12, 52 weeks)*
    - *fasting lipids, insulin and glucose (0, 12, 52 weeks)*
    - *fasting folate, homocysteine, C reactive protein (0, 12, 52 weeks)*

***VITAMIN B12 STATUS:*** *(0, 12, 52 weeks)*

- *Plasma folate and B12*
- *Homocysteine*

***DIETARY ASSESSMENT***

- *Food Frequency Questionnaire (0, 6months, 12 months)*
- *Three day weighed food records (weeks 2,4,6,8,10,12)*
- *Daily Checklists (first 12 weeks only)*

***PHYSICAL PERFORMANCE/EXERCISE TOLERANCE***

- *exercise tolerance assessment (0, 12, 52 weeks)*

*(aerobic capacity, local muscular strength, power and endurance)*

***PSYCHOLOGICAL WELLBEING***

- - - *assessment of self esteem, self efficacy, quality of life, (0, 12, 52 weeks)*
    - *Mood assessed at baseline and every 2 weeks for 12 weeks and monthly until week 52*
    - *assessment of barriers, enhancers to maintaining longer term lifestyle changes (at each monthly visit)*
    - *subjective assessment of diet – palatability, ease of adherence etc (week 12)*

***SEXUAL FUNCTION***

- *sexual function assessed by questionnaires (0, 12, 52 weeks)*
- IIEF-15
- LUTS
- SHBG, testosterone and BT

***SAFETY MARKERS***

- - - *fasting creatinine (0, 12, 52 weeks)*
    - *24hr urine samples for urea/creatinine, markers of bone turnover, sodium (0, 12, 52 weeks)*

***CONTROLLING VARIABLES***

- - - *measurement of exercise – physical activity diary (0, 12, 52 weeks)*
    - *measurement of usual food intake at baseline using FFQ*

**STUDY FLOWCHART**

| **Week** | **screening** | **-1** | **0** | **2** | **4** | **6** | **8** | **10** | **12** | **16-48** | **52** |
| --- | --- | --- | --- | --- | --- | --- | --- | --- | --- | --- | --- |
| **Visits** | **VS** | **V0** | **V1V2** | **V3** | **V4** | **V5** | **V6** | **V7** | **V8V9** | **V10-19** | **V20V21** |
| Time needed | 15min | 30min | 2hr each | 30min | 30min | 30min | 30min | 30min | 2hr ea | 30min | 2hr ea |
| Height (once only) | X |  |  | X | X | X | X | X | XX | X | X |
| Weight and waist circumference | X |  | XX | X | X | X | X | X | XX | X | X |
| Faecal sample (48hr) |  |  | X |  |  |  |  |  | X |  | X |
| Rectal Biopsy |  |  | X |  |  |  |  |  | X |  | X |
| Blood Pressure | X |  | XX |  |  |  |  |  | XX |  | XX |
| Fasting blood sample | X |  | XX |  |  |  |  |  | XX |  | XX |
| DEXA |  | X |  |  |  |  |  |  | X |  | X |
| Psychological assessment |  |  | X |  |  |  |  |  | X |  | X |
| Mood |  |  | X |  | X |  | X |  | X |  | X |
| Sexual function/desire questionnaires |  |  | X |  |  |  |  |  | X |  | X |
| Physical activity diary |  | X |  |  |  |  |  |  | X |  | X |
| Graded treadmill test |  | X |  |  |  |  |  |  | X |  |  |
| Muscular strength, power and  endurance tests |  | X |  |  |  |  |  |  | X |  |  |
| Diet checklist (daily) |  |  | X | X | X | X | X |  |  |  |  |
| Food frequency questionnaire |  |  | X |  |  |  |  |  |  | X 6mo | X |
| Diet data entry (3 days) |  |  |  | X | X | X | X | X | X |  |  |
